# Supplementary material for: Targeted metabolomics study of serum bile acid profile in patients with end-stage renal disease undergoing hemodialysis
Source: PeerJ. 2019 Jun 17;7:e7145. doi: 10.7717/peerj.7145 (PMC6585905; doi:10.7717/peerj.7145)
Supplement: Supplemental Information 1 [file peerj-07-7145-s001.docx]

**Targeted metabolomics study of serum bile acid profile in patients with end-stage renal disease undergoing hemodialysis**

**SUPPLEMENTARY INFORMATION**

**Supplementary table 1 Parameters for quantiﬁcation on 26 bile acids by UPLC-MS/MS**

| Compounds | RetentionTime  (min) | Transition  (m/z) | DP  (V) | CE  (eV) | linerrange  (ng/mL) | CalibrationCurves* | *R*^2^ | Internalstandard |
| --- | --- | --- | --- | --- | --- | --- | --- | --- |
| CA | 4.00 | 407>407 | -220 | -20 | 1-200 | y=0.01417x+0.00760 | 0.9999 | CA-d_4_ |
| CDCA | 4.77 | 391>391 | -220 | -20 | 1-200 | y=0.01879x-0.00976 | 0.9995 | CDCA-d_4_ |
| DCA | 4.89 | 391>391 | -220 | -20 | 1-100 | y=0.01830x-0.00104 | 0.9996 | DCA-d_4_ |
| HDCA | 3.82 | 391>391 | -220 | -20 | 1-50 | y=0.110183x+0.000776 | 0.9974 | GCA-d_4_ |
| LCA | 5.41 | 375>375 | -220 | -20 | 1-50 | y=0.03199x-0.01099 | 0.9992 | LCA-d_4_ |
| UDCA | 3.58 | 391>391 | -220 | -20 | 1-50 | y=0.0976x+0.0734 | 0.9997 | UDCA-d_4_ |
| α+ωMCA | 3.07 | 407>407 | -220 | -20 | 1-100 | y=0.00896x+0.00246 | 0.9997 | CA-d_4_ |
| βMCA | 3.15 | 407>407 | -220 | -20 | 1-50 | y=0.01165x+0.00861 | 0.9988 | CA-d_4_ |
| γMCA | 3.61 | 407>407 | -220 | -20 | 1-100 | y=0.00921x+0.00249 | 0.9998 | CA-d_4_ |
| 7KLCA | 3.75 | 389>389 | -220 | -20 | 1-50 | y=0.0279x-0.0128 | 0.9993 | LCA-d_4_ |
| 12KLCA | 3.88 | 389>389 | -220 | -20 | 1-50 | y=0.01742x-0.00848 | 0.9992 | LCA-d_4_ |
| 6,7-diketoLCA | 2.26 | 403>403 | -220 | -20 | 1-50 | y=0.013028x-0.001399 | 0.9993 | LCA-d_4_ |
| GCA | 3.20 | 464>74 | -180 | -75 | 2-500 | y=0.0227x-0.0249 | 0.9998 | GCA-d_4_ |
| GCDCA | 3.85 | 448>74 | -180 | -75 | 5-200 | y=0.01891x-0.00153 | 0.9994 | GCDCA-d_4_ |
| GDCA | 4.02 | 448>74 | -180 | -75 | 1-100 | y=0.01768x-0.00415 | 0.9996 | GDCA-d_4_ |
| GHCA | 2.71 | 464>74 | -180 | -75 | 1-100 | y=0.02145x-0.00441 | 0.9993 | GCA-d_4_ |
| GLCA | 4.62 | 432>74 | -180 | -75 | 1-50 | y=0.0248x+0.0036 | 0.9996 | GLCA-d_4_ |
| GUDCA | 2.73 | 448>74 | -180 | -75 | 5-500 | y=0.0265x-0.0441 | 0.9991 | GUDCA-d_4_ |
| TCA | 3.11 | 514>80 | -150 | -140 | 2-200 | y=0.02794x-0.01604 | 0.9998 | GCA-d_4_ |
| TCDCA | 3.74 | 498>80 | -150 | -140 | 2-200 | y=0.03042x-0.00582 | 0.9992 | GCDCA-d_4_ |
| TDCA | 3.93 | 498>80 | -150 | -140 | 1-100 | y=0.03463x-0.00908 | 0.9989 | GCA-d_4_ |
| THCA | 2.64 | 514>80 | -150 | -140 | 1-100 | y=0.02997x-0.00825 | 0.9996 | GCA-d_4_ |
| TαMCA | 2.24 | 514>80 | -150 | -140 | 1-50 | y=0.01708x+0.00211 | 0.9986 | GCA-d_4_ |
| TβMCA | 2.29 | 514>80 | -150 | -140 | 1-100 | y=0.0290x-0.0172 | 0.9977 | GCA-d_4_ |
| TUDCA | 2.68 | 498>80 | -150 | -140 | 1-50 | y=0.0269x-0.0147 | 0.9958 | GCA-d_4_ |

* y, the integral peak area ratio between standard and IS (internal standard); x, concentration in the detected samples or standard curves samples.
